# Genetic ablation of homeodomain-interacting protein kinase 2 selectively induces apoptosis of cerebellar Purkinje cells during adulthood and generates an ataxic-like phenotype

**DOI:** 10.1038/cddis.2015.298

**Published:** 2015-12-03

**Authors:** S Anzilotti, M Tornincasa, R Gerlini, A Conte, P Brancaccio, O Cuomo, G Bianco, A Fusco, L Annunziato, G Pignataro, G M Pierantoni

**Affiliations:** 1 SDN IRCCS, Naples, Italy; 2 Institute of Endocrinology and Experimental Oncology of National Research Council and Department of Molecular Medicine and Medical Biotechnology, School of Medicine, ‘Federico II' University of Naples, Naples, Italy; 3 Division of Pharmacology, Department of Neuroscience, Reproductive and Dentistry Sciences, School of Medicine, ‘Federico II' University of Naples, Naples, Italy

## Abstract

Homeodomain-interacting protein kinase 2 (HIPK2) is a multitalented coregulator of an increasing number of transcription factors and cofactors involved in cell death and proliferation in several organs and systems. As *Hipk2*^*−/−*^ mice show behavioral abnormalities consistent with cerebellar dysfunction, we investigated whether Hipk2 is involved in these neurological symptoms. To this aim, we characterized the postnatal developmental expression profile of Hipk2 in the brain cortex, hippocampus, striatum, and cerebellum of mice by real-time PCR, western blot analysis, and immunohistochemistry. Notably, we found that whereas in the brain cortex, hippocampus, and striatum, HIPK2 expression progressively decreased with age, that is, from postnatal day 1 to adulthood, it increased in the cerebellum. Interestingly, mice lacking Hipk2 displayed atrophic lobules and a visibly smaller cerebellum than did wild-type mice. More important, the cerebellum of *Hipk2*^*−/−*^ mice showed a strong reduction in cerebellar Purkinje neurons during adulthood. Such reduction is due to the activation of an apoptotic process associated with a compromised proteasomal function followed by an unpredicted accumulation of ubiquitinated proteins. In particular, Purkinje cell dysfunction was characterized by a strong accumulation of ubiquitinated *β*-catenin. Moreover, our behavioral tests showed that *Hipk2*^*−/−*^ mice displayed muscle and balance impairment, indicative of Hipk2 involvement in cerebellar function. Taken together, these results indicate that Hipk2 exerts a relevant role in the survival of cerebellar Purkinje cells and that *Hipk2* genetic ablation generates cerebellar dysfunction compatible with an ataxic-like phenotype.

Homeodomain-interacting protein kinases (HIPKs), which include HIPK1/2/3/4 (HIPK1–4), are members of a family of nuclear serine/threonine kinases that affect cell proliferation, differentiation, and apoptosis^1^ by influencing gene transcription. Among these kinases, HIPK2 appears to exert multiple functions depending on the phosphorylation of the downstream target proteins. In effect, HIPK2 modulates cell fate in different ways: for example, it decreases cellular proliferation and survival by modulating the Wnt/*β*-catenin pathway,^2, 3^ and it promotes cell death by phosphorylating and activating the pro-apoptotic factor p53.^1^ Indeed, Hipk2 knockdown increases the stability of *β*-catenin and its accumulation in the nucleus, thus enhancing the expression of Wnt target genes and cell proliferation.^2^ However, *Hipk2* genetic ablation can also impair proliferation in different cell types including fetal liver cells,^3^ mouse embryo fibroblasts (MEFs),^4, 5^ bone marrow,^6^ and sensory neurons.^7^

Zhang *et al.*^8^ observed that Hipk2^*−/−*^ mutants show a severe psychomotor behavioral phenotype that reflects neuronal deficiencies in the substantia nigra pars compacta and the ventral tegmental area,^9^ and we (personal and unpublished observations) noticed that *Hipk2* knockout mice show behavioral abnormalities consistent with cerebellar defects, such as dystonia, impaired coordination, reduced motility, and clasping of posterior limbs.^8^ Thus, on the basis of these observations, we investigated whether Hipk2 is involved in cerebellar functions.

To this aim, we first characterized the postnatal developmental expression profile of Hipk2 in the cerebellum, brain cortex, hippocampus, and striatum of mice by real-time PCR, western blot analysis, and immunohistochemistry. Then, we examined the cerebellum of Hipk2^*−/−*^ mice at the cellular level and the behavior of these animals in terms of cerebellar dysfunction.

## Results

### *Hipk2* is ubiquitously expressed in the brain and increases with age in the cerebellum whereas it decreases in other cerebral regions

**Figure 1:**
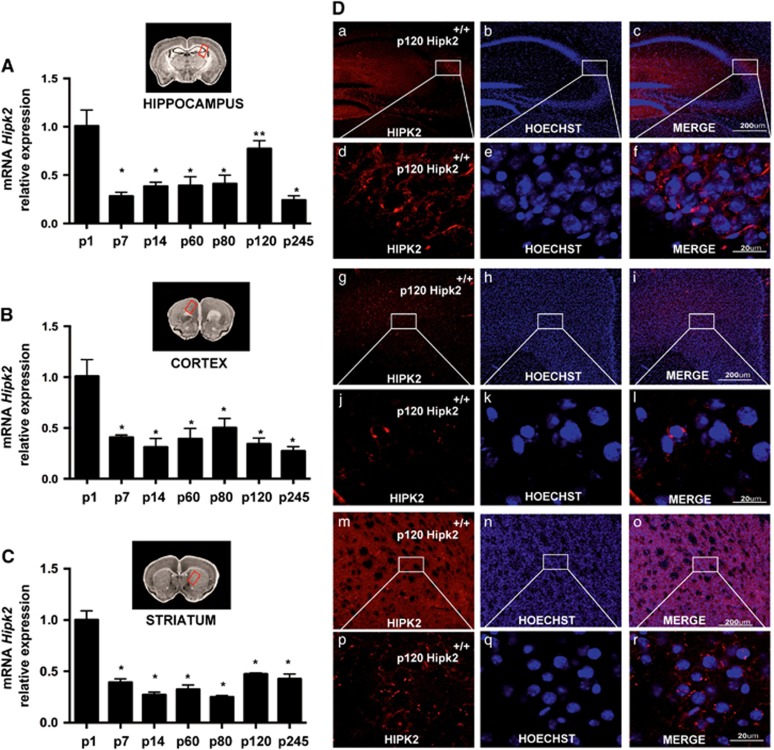
*Hipk2* expression in hippocampus, cortex, and striatum of wild-type mice. RNA extracted from hippocampus (**A**), cortex (**B**), and striatum (**C**) of wild-type mice at different ages was analyzed by qRT-PCR for *Hipk2* expression. The *G6pd* expression level was used for normalization. Two-way repeated-measures ANOVA *P*<0.001; *P<0.001 *versus* p1, **P<0.001 *versus* all time points, *n*=3 for each group of age. HIPK2 expression in different brain regions (**D**). Double labeling of HIPK2 and Hoechst in hippocampus (**D** a–f), cortex (**D** g–l), and striatum (**D** m–r) by confocal microscopy analysis. Scale bar, 200 *μ*m. High magnification of a portion of hippocampus (**D** d–f), cortex (**D** j–l), and striatum (**D** p–r). Scale bar, 20 *μ*m

**Figure 2:**
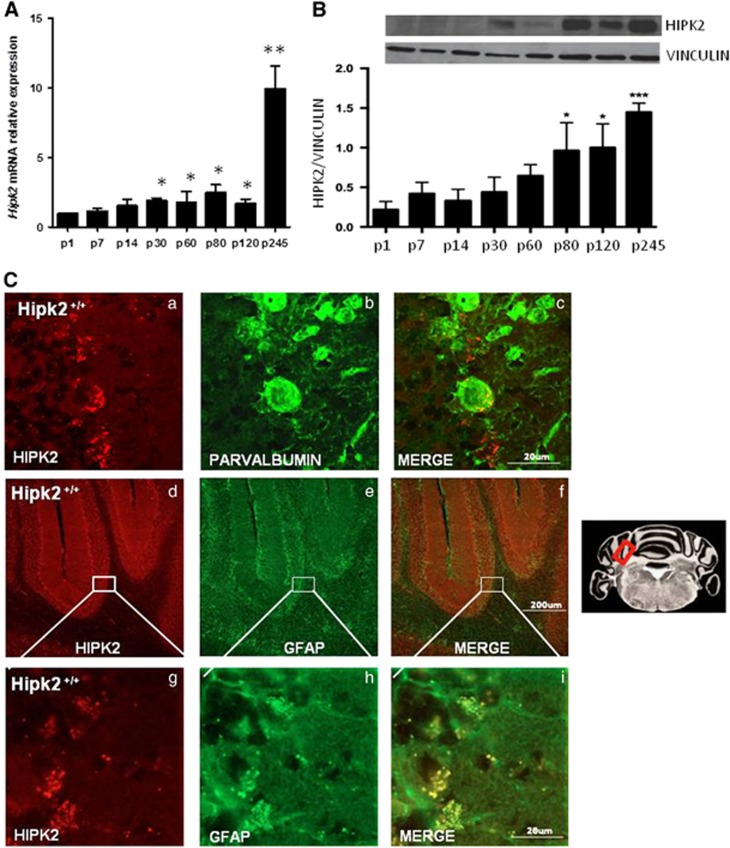
HIPK2 expression in cerebellum. (**A**) RNA extracted from cerebellum of wild-type mice at different ages was analyzed by qRT-PCR for *Hipk2* expression. The *G6pd* expression level was used for normalization. ANOVA test was used for statistical analysis. Data represent the mean±S.E.M. *Significant difference (*P*<0.05) at given time points, *n*=6 for each age groups. (**B**) Proteins extracted from cerebellum of wild-type mice were analyzed by western blot analysis for HIPK2 expression. Vinculin was used for normalization. Densitometric analysis of three independent experiments is shown. Student's *t*-test, *n*=3 for each group. Data represent the mean±S.D. *P<0.05 and ***P<0.001. One representative of three independent experiments is shown. Confocal images from cerebellar cortex sections of p120 wild-type mice performed with anti-HIPK2 (**C** a), anti-Parvalbumin (**C** b) antibodies, and merged images (**C** c). Scale bars, 20 *μ*m. Immunofluorescence staining performed with anti-HIPK2 (**C** d) and anti-GFAP antibodies (**C** e) and Merge (**C** f) in cerebellar cortex sections from p120 wild-type mice. Scale bars, 200 *μ*m. High magnification of HIPK2 (**C** g), GFAP (**C** h), and merge (**C** i). Scale bars, 20 *μ*m. **P<0.01

HIPK2 expression was evaluated in the brain cortex, hippocampus, striatum, and cerebellum of wild-type mice at different ages, from postnatal day 1 (p1) to postnatal day 245 (p245), by real-time RT-PCR, western blot analysis, and immunohistochemistry (Figures 1 and 2). *Hipk2* expression decreased in the hippocampus, cortex, and striatum during the first postnatal week and remained stable thereafter (Figures 1A–C). In contrast, in the cerebellum it strongly increased with age at both the mRNA (Figure 2A) and protein expression levels (Figure 2B). In particular, *HIPK2* mRNA expression increased at p30 and p80 and peaked at p245 (Figure 2A); similarly, HIPK2 protein expression increased at p80 and p120 and peaked at p245 (Figure 2B).

Confocal microscopy quantified HIPK2 cellular localization in hippocampal, cortical, striatal, and cerebellar coronal brain slices of p120-old mice (Figure 1D). Single-label immunohistochemical studies confirmed that HIPK2 was highly expressed in the neuronal perikarya of all examined brain regions (Figure 1D: hippocampus, a–f; cortex, g–l; striatum, m–r). In particular, HIPK2 was significantly expressed in the primary, secondary motor, and cingulated cortices, whereas it was weakly expressed in the remaining area of this region. In the hippocampus, HIPK2 was preferentially expressed in the CA2 and CA3 regions, whereas it was almost undetectable in both the CA1 region and the dentate gyrus. In the striatum, HIPK2 was principally expressed in the caudato putamen area. In the cerebellum, HIPK2 was detected mainly at the level of the Purkinje cell layer (Figure 2C). In this layer, HIPK2 colabeled with parvalbumin, a specific marker of Purkinje cells and molecular layer interneurons (Figures 2C a–c), in some Purkinje cell somata and with glial fibrillary acid protein (GFAP), a marker of Bergman glia somata, in some astrocytes (Figures 2C d–i). Notably, in all the examined brain regions, HIPK2 localized mainly in the cytoplasm.

### *Hipk2* genetic ablation is associated with cerebellum lobular atrophy and Purkinje cell apoptosis

**Figure 3:**
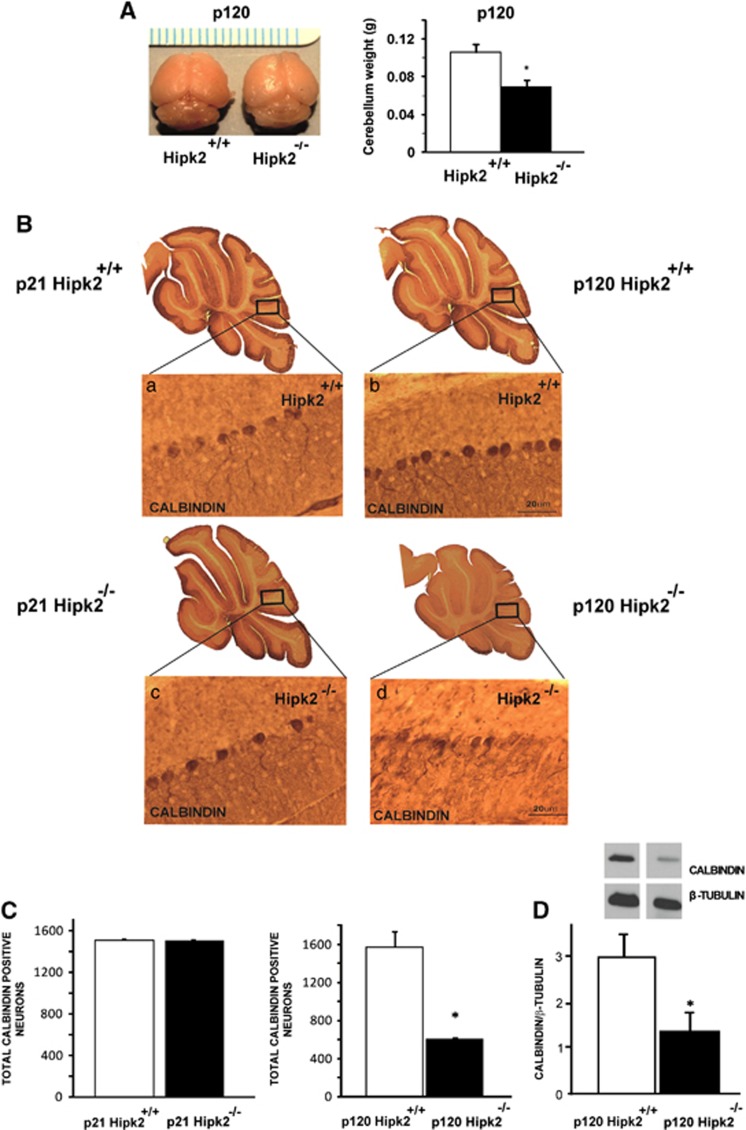
Absence of HIPK2 reduces Purkinje cells in cerebellum. (**A**) Macroscopic appearance of P120 wild-type and *Hipk2*^*−/−*^ mice. Cerebellum weight of wild-type and *Hipk2*^*−/−*^ mice at P120. Student's *t*-test, *n*=3 for each genotype. Data represent the mean±S.D. *P<0.05. (**B**) Representative image of calbindin 3,3′-diaminobenzidine (DAB) staining of wild-type mice and *Hipk2*^*−/−*^ mice at P21 and P120. Magnified views of DAB staining of wild type at P21 (a); *Hipk2*^*−/−*^ mice at P21 (c); wild-type mice at P120 (b); calbindin DAB staining of *Hipk2*^*−/−*^ mice at P120 (d). (**C**) Cell-counting analysis of calbindin-positive neurons in the cerebellum of P21 and P120 wild-type and *Hipk2*^*−/−*^ mice. Student's *t*-test, *n*=3 for each genotype. Data represent the mean±S.D. *P<0.05. (**D**) Representative image of western blot of total extract proteins from cerebellum, performed with anti-calbindin antibodies, is shown. Densitometric analysis of western blot analysis performed with calbindin antibodies is reported. For statistical analysis, Student's *t*-test, *n*=3 for each genotype. Data represent the mean±S.D. *P<0.05

The role played by HIPK2 in the cerebellum was further investigated through the use of *Hipk2*^*−/−*^ mice (Supplementary Figure 1). Interestingly, the cerebella of *Hipk2*^*−/−*^ mice (p120) were visibly smaller than those of wild-type mice (Figure 3A). Moreover, immunohistochemical studies, with antibodies against calbindin, a specific marker of Purkinje cells, revealed pronounced atrophy of cerebellar lobules in *Hipk2*^*−/−*^ adult mice (p120) (Figure 3B). This phenomenon was associated with an alteration in Purkinje layers. Indeed, Purkinje cells underwent morphological alterations and a degeneration of dendritic branching (Figure 3B b and d). Furthermore, to evaluate whether these cerebellar abnormalities were due to alterations in cerebellar development or to a degenerative process, we also analyzed the cerebella of juvenile *Hipk2*^*−/−*^ mice (p21), the stage in which the cerebellum is completely developed. No differences between wild-type and *Hipk2*^*−/−*^ mice were observed at this age in terms of Purkinje cell architecture, morphology, and dendritic arborization (Figures 3B a–c), thus suggesting a degenerative process. Moreover, counting analysis of calbindin-positive cells showed a strong reduction in Purkinje cells in *Hipk2*^*−/−*^ at p120 (Figure 3C), whereas at p21 the number of Purkinje cells did not change (Figure 3C). These results were further confirmed by western blot analysis of calbindin expression (Figure 3D). Interestingly, no differences in the number of other neuron cell types were found between wild-type and *Hipk2*^*−/−*^ mice (Supplementary Figure 2).

### Genetic ablation of *Hipk2* induces apoptosis of Purkinje cells

**Figure 4:**
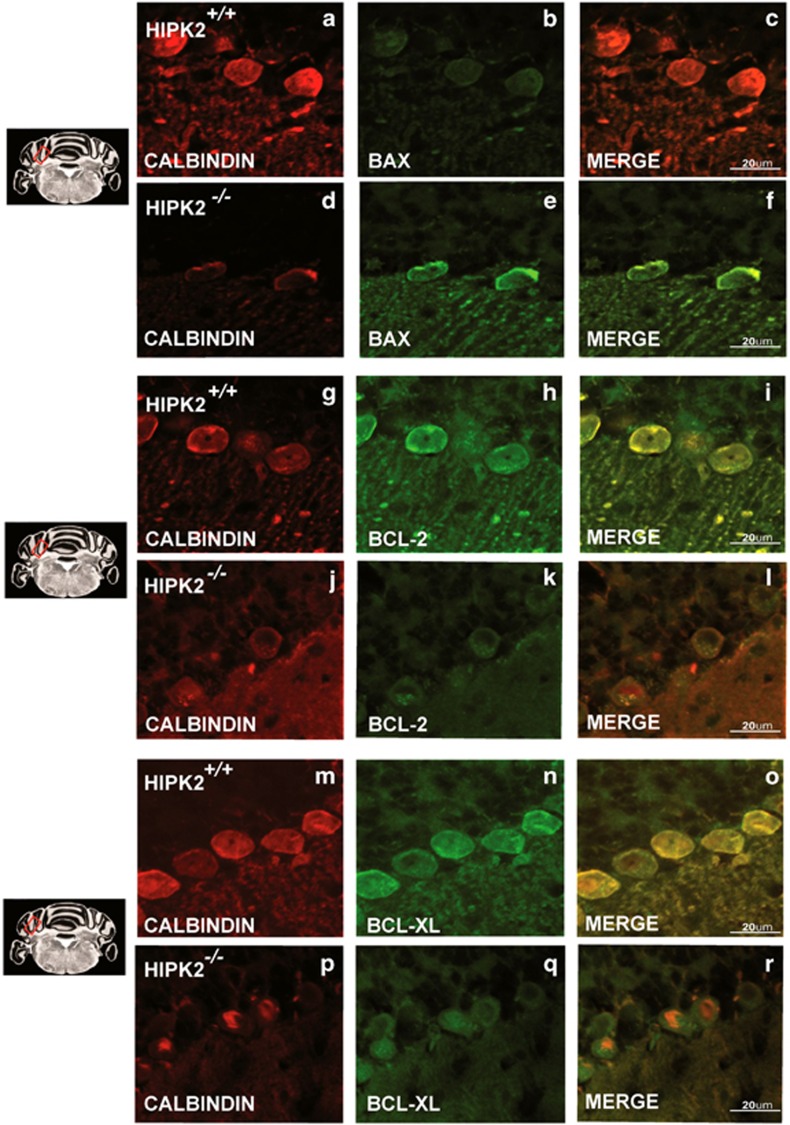
The absence of HIPK2 induces an activation of pro-apoptotic pathways. Confocal images of cerebellar cortex sections from wild-type (**a–c**, **g–i,** and **m–o**) and *Hipk2*^*−/−*^ mice (**d–f**, **j–l**, and **p–r**) performed with antibodies against BAX (**b** and **e**), BCL-2 (**h** and **k**), BCL-XL (**n** and **q**), and calbindin (**a–p**), and merged images (**c–r**). Scale bar, 20 *μ*m. All pictures were taken in the same conditions

To investigate the mechanism responsible for the reduction of Purkinje cells in *Hipk2*^*−/−*^ mice, we performed immunofluorescence analysis on coronal cerebellar cortex sections from p120 wild-type and *Hipk2*^*−/−*^mice. For this study, we used antibodies directed against the pro-apoptotic factor BAX and the anti-apoptotic factors BCL-2 and BCL-XL. In *Hipk2*^*−/−*^ calbindin-positive neurons, BAX expression increased (Figures 4d–f), whereas BCL-2 (Figures 4j–l) and BCL-XL (Figures 4p–r) expression decreased, as compared with wild-type mice (Figures 4a–c, g–i, m–o). Presumably, these alterations may have elicited the activation of caspases observed in the Purkinje cells of *Hipk2*^*−/−*^ mice (data not shown). Interestingly, although the role of HIPK2 in apoptosis is often related to p53 activity,^1^ no differences in p53 expression were found between wild-type and *Hipk2*^*−/−*^ Purkinje neurons (data not shown).

### Genetic ablation of *Hipk2* produces accumulation of ubiquitinated proteins, such as *β*-catenin, in Purkinje cells

**Figure 5:**
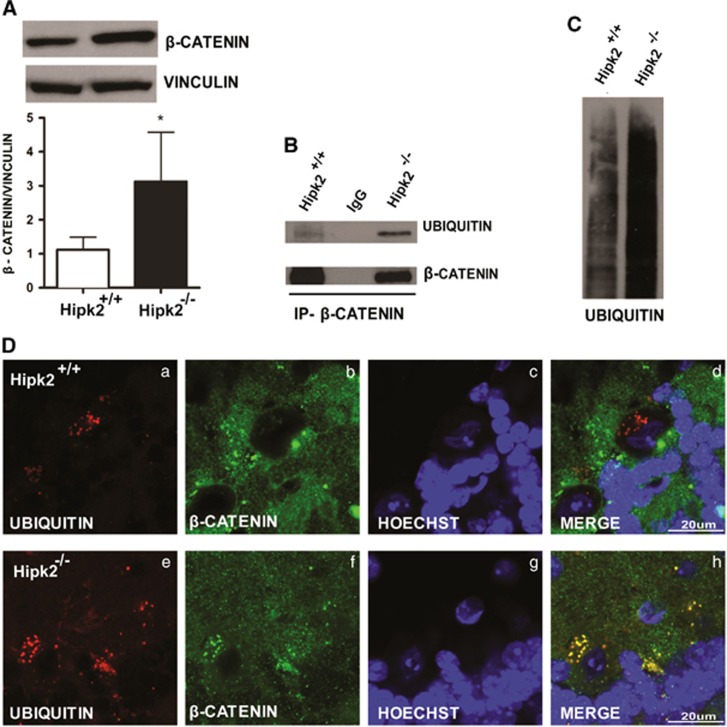
Characterization of *β*-catenin pathway in the cerebellum of *Hipk2*^*−/−*^ mice. (**A**) *β*-Catenin protein expression levels evaluated in total extracts of cerebellum from wild-type and *Hipk2*^*−/−*^ mice by western blot analysis. Vinculin was used for normalization. One representative experiment is shown. Four samples of wild-type and five samples of *Hipk2*^*−/−*^ were used for this analysis. For statistical analysis, Student's *t*-test was used. Data represent the mean±S.D. *P<0.05. (**B**) Total extracts from the cerebellum of wild-type and *Hipk2*^*−/−*^ mice were immunoprecipitated using anti-*β*-catenin antibody and analyzed by western blot with anti-ubiquitin and anti-*β*-catenin antibodies. Polyclonal rabbit IgG was used as negative control. (**C**) Western blot analysis performed with total extracts from the cerebellum of wild-type and *Hipk2*^*−/−*^ mice using anti-ubiquitin antibodies. One representative experiment is shown. Three samples of wild-type mice and three samples of *Hipk2*^*−/−*^ mice were used. Confocal images of cerebellar cortex sections from wild-type (**D** a–d) and *Hipk2*^*−/−*^ mice (**D** e–h) performed with antibodies against ubiquitin (a and e), *β*-catenin (b and f), and Hoechst (c and g), and merged images (d and h). Scale bar, 200 *μ*m

As HIPK2 acts as a negative regulator of the Wnt/*β*-catenin pathway and its knockdown elicits an accumulation of *β*-catenin in the nucleus,^2^ we investigated whether this pathway was also dysregulated in the cerebellar Purkinje neurons of *Hipk2*^*−/−*^ mice. *β*-Catenin expression was increased in total protein extracts from the cerebellum of *Hipk2*^*−/−*^ mice compared with wild-type mice (p120) (Figure 5A). Furthermore, because *β*-catenin accumulation may be due to a deficit in its proteasomal degradation,^10^ we performed double immunofluorescence analysis using anti-ubiquitin and anti-*β*-catenin antibodies. This analysis showed that *β*-catenin completely colocalized with ubiquitin in *Hipk2*^*−/−*^ but not in wild-type mice (Figure 5D d and h). Surprisingly, although *β*-catenin colocalized with ubiquitin, it was not degraded. To confirm that *β*-catenin ubiquitination was higher in *Hipk2*^*−/−*^ cerebella than in wild-type cerebella, total protein extracts from both wild-type and *Hipk2*^*−/−*^ cerebella were immunoprecipitated with an anti-*β*-catenin antibody and then revealed with an anti-ubiquitin antibody (Figure 5B). *β*-Catenin was much more ubiquitinated in *Hipk2*^*−/−*^ than in wild-type cerebellum. Furthermore, western blot analysis with anti-ubiquitin antibodies on total cerebellar protein extracts revealed that the total amount of ubiquitinated proteins increased in *Hipk2*^*−/−*^ mice compared with wild-type mice (Figure 5C). This suggests that the axis ubiquitin–proteasome may be severely impaired in *Hipk2*^*−/−*^ cerebella.

### Genetic ablation of *Hipk2* causes astrogliosis in the cerebellum

**Figure 6:**
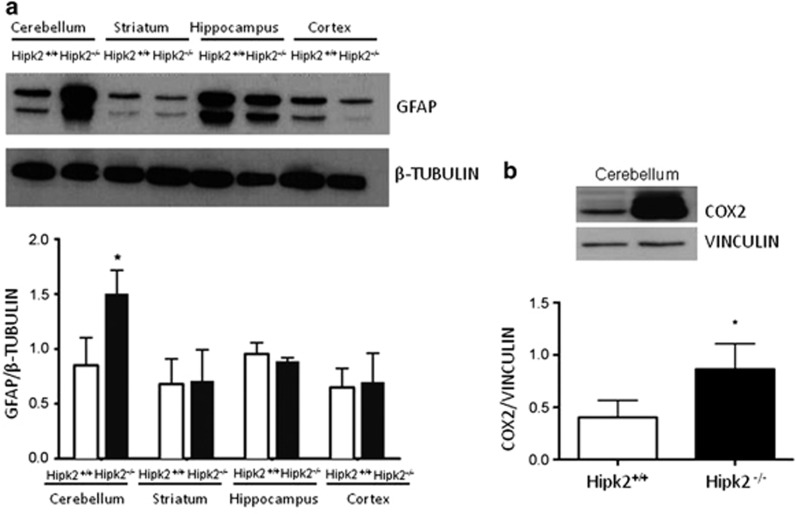
Lack of HIPK2 induces a strong increase in astroglial cells. (**a**) Western blot analysis performed using GFAP antibodies of proteins extracted from cerebellum, striatum, hippocampus, and cortex. One representative experiment is shown. *β*-Tubulin was used for normalization. Densitometric analysis of three independent experiments is shown. Student's *t*-test, *n*=3 for each genotype. Data represent the mean±S.D. *P<0.05. (**b**) Western blot analysis performed with total extracts from the cerebellum of wild-type and *Hipk2*^*−/−*^ mice using COX2 antibody. Vinculin was used for normalization. One representative experiment is shown. Densitometric analysis of western blot was performed using COX2 antibodies. For this analysis, four samples of wild-type mice and four samples of *Hipk2*^*−/−*^ were used. For statistical analysis, Student's *t*-test was used for each genotype. Data represent the mean±S.D. *P<0.05

Immunohistochemical results showed that HIPK2 was expressed in neurons and in glial cells characterized by small somata extending radial fibers toward the pial surface with numerous collateral branches and in stellate-shaped astrocytes (Figures 2d–i). These glia were identified as Bergmann glia and astroglia using the specific marker GFAP. To evaluate whether GFAP protein expression was modified in the absence of HIPK2, we performed western blot analysis in the cerebellum, cortex, hippocampus, and striatum of *Hipk2*^*−/−*^ and wild-type mice. GFAP protein expression was significantly higher in the cerebellum of *Hipk2*^*−/−*^ mice as compared with wild-type animals. Intriguingly, this increase was evident only in the cerebellar cortex and not in the other brain regions examined (Figure 6a). In addition, as the increase in GFAP-positive neuronal cells is generally associated with inflammation and astrogliosis,^9, 11^ we evaluated the expression of cyclooxygenase-2 (COX 2). As expected, the expression of this inflammatory marker increased in the cerebellum of *Hipk2*^*−/−*^ mice, suggesting a correlation between astrogliosis and inflammation (Figure 6b).

Finally, to verify whether the ablation of HIPK2 could also affect other types of glial cells in the cerebellum, we performed western blot analysis using antibodies against Iba1, a specific marker of microglia. No significant differences were observed between wild-type and *Hipk2*^*−/−*^ mice (Supplementary Figure 2A). Altogether, these findings suggest that the degeneration of Purkinje cells in *Hipk2*^*−/−*^ adult mice is associated with a pronounced increase of astroglial cells.

### *Hipk2*^*−/−*^ mice exhibit altered motor coordination and muscle impairment

**Figure 7:**
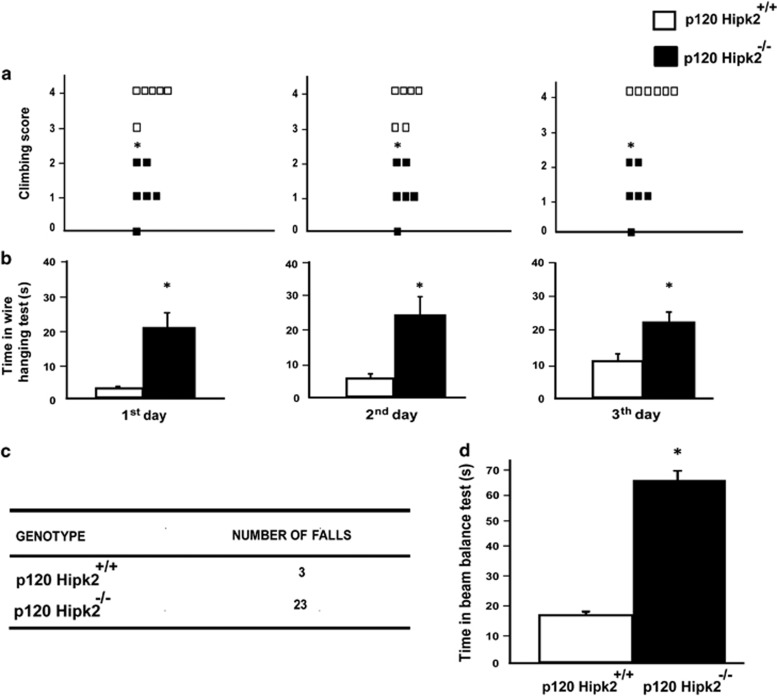
Muscle and motor impairment in *Hipk2*^*−/−*^ mutants. (**a**) Six wild-type and six *Hipk2*^*−/−*^ mice at P120 were subjected to wire hanging test: score from 0 to 5 (5 being the highest; 0: fell off; 1: hung onto the bar with two forepaws; 2: in addition to 1, attempted to climb onto the bar; 3: hung onto the bar with both forepaws and one or both hindpaws; 4: hung onto the bar with all four paws with tail wrapped around the bar; and 5: escaped to one of the supports). During the 3-day training, five of *Hipk2*^*−/−*^ mice had a score between 1 and 2, whereas one always fell. Nonparametric Kruskal–Wallis test, *n*=6 for each genotype. (**b**) Time to reach the right position in the wire hanging test. One-way ANOVA *P*=0.0000, *n*=6 mice for each genotype. (**c**) The same mice were analyzed by the beam balance test. The table reports the number of falls in the 3-day training. (**d**) The graph shows the traveling time on the beam. The average time of three trials per beam for three consecutive days of experiments is indicated. One-way ANOVA *P*=0.000, *n*=6 mice for each group. Data represent the mean±S.D. *P<0.01

As *Hipk2*^*−/−*^ mice displayed a reduction in Purkinje cells, which are mostly responsible for motor coordination and cerebellum-mediated balance,^12^ we assessed the motor behavior of six wild-type and six *Hipk2*^*−/−*^ mice by wire hanging and beam balance tests. Because symptoms of motor dysregulation were clearly evident with increasing age, we analyzed only mice at p120. In brief, in the wire hanging test, which measures muscle impairment, each mouse received a climbing score ranging from 1 to 5 (5 being the highest). We observed consistent differences between wild-type and *Hipk2*^*−/−*^ mice. Wild-type mice had an average score of 4, whereas *Hipk2*^*−/−*^ mice had an average score of 1. Moreover, *Hipk2*^*−/−*^ mutants frequently fell because of difficulties in hanging onto the wire (Figure 7a). Notably, one mouse was totally unable to climb (Figure 7a). To evaluate whether the animals learned the motor schemes of movement, the test was repeated for three consecutive days. *Hipk2*^*−/−*^ mice always repeated the same wrong movements, and the score for each mouse did not change throughout the entire experiment. These mice also manifested forelimb dystonia. Indeed, the mean hanging time of *Hipk2*^*−/−*^ was significantly greater than that of wild-type mice (Figure 7b).

Motor coordination was then evaluated with the beam balance test. This test showed that *Hipk2*^*−/−*^ mice displayed akinesia, postural instability, and poor coordination. Indeed, the number of falls during the 3-day experimental period was significantly higher in *Hipk2*^*−/−*^ than in wild-type mice (Figure 7c). Furthermore, *Hipk2*^*−/−*^ mice were much slower than the wild-type in covering the same distance (50 cm; 60 *versus* 20 s, respectively; Figure 7d). Taken together, these results highlight a potential correlation between cerebellar alterations and impaired motor behavior (awkward gait and muscle weakness) in *Hipk2*^*−/−*^ mice.

## Discussion

In the present study, we demonstrated for the first time that the kinase *HIPK2* is relevant for Purkinje cell survival. Indeed, the cerebellum of *Hipk2*^*−/−*^ mice appeared visibly smaller than that of wild-type mice and displayed atrophic lobules. More important, we found a strong reduction in the number of cerebellar Purkinje neurons because of the activation of an apoptotic process associated with a compromised ubiquitin-mediated proteasomal degradation pathways. In particular, Purkinje cell dysfunction in *Hipk2*^*−/−*^ mice was characterized by a strong accumulation of ubiquitinated *β*-catenin. Interestingly, these cellular modifications because of the genetic ablation of *Hipk2* gave rise to a phenotype characterized by muscle and balance impairment suggesting *Hipk2* involvement in cerebellar functions. Indeed, similar to other Hipk2 knockout mice described by other authors,^8^ these Hipk2-null mice showed strong dystonia characterized by the clasping of hind limbs when mice were suspended by their tails, consistent failure to finish the tandem walk, poor motor coordination, and reduced responses to novelty. In addition, *HIPK2*-null mice display phenotypic differences. In particular, p30 *HIPK2*-null mice were significantly smaller than their wild-type counterparts. Moreover, *HIPK2*-null mice are characterized by head and snout crushed, a curvature of the spine, and distortion of the front and rear legs (data not shown).

All these findings add further insights into the role of *Hipk2* in the central nervous system (CNS). Previously research carried out by Zhang *et al.*^8^ has shown that HIPK2 is involved in transforming growth factor-*β* (TGF*β*)-dependent survival of midbrain dopamine neurons, and its genetic ablation induces extrapyramidal defects in mice.^8^ Similarly, we have recently demonstrated that in nonneuronal cell lines, such as MEF cells, HIPK2 regulates cytokinesis – the last step of the cell cycle – and *Hipk2* depletion results in cytokinesis failure and tetraploidization.^5, 13^ Consistently, HIPK2, working in tandem with Pin1 (peptidyl-prolylcis-trans isomerase NIMA-interacting), promotes brain cortex neurogenesis.^14^

In contrast with these findings, another report states that in *Hipk2*^*−/−*^ mice neuronal defects are correlated with an upregulation of the transcription factor Brn3a and of its target genes in the trigeminal ganglion, a phenomenon that eventually leads to reduced apoptosis and increased neurogenesis.^7^ This apparent discrepancy might though be explained by the different cell population examined and suggests a peculiar cell-specific role for HIPK2.

An aspect that needs to be underlined is that the reduction of Purkinje cells in the cerebellum of *Hipk2^−/−^* mice was accompanied by cerebellar astrogliosis and increased expression of both GFAP and COX-2, the latter being a marker of astrocyte-mediated inflammatory response.^12^ Notably, the astroglial response in *Hipk*2^*−/−*^ mice was selective for the cerebellum as GFAP upregulation was not detected in any of the other brain regions examined.

Several molecular mechanisms responsible for the cerebellar alterations observed in the present study have been proposed, and the role of HIPK2 in cell development has also been highlighted in several studies.^15, 16, 17^ Regarding the molecular mechanisms, some studies point out that the VEGF,^18^ TGF*β*,^8^ and Wnt/*β*-catenin pathways cooperate with HIPK2 in cell development. For instance, the Wnt/*β*-catenin signaling pathway plays an important role in animal development during embryogenesis^19^ and is important for the development of cortical and hippocampal neurons.^20^ Evidence of the correlation between this pathway and *Hipk2* is that the genetic ablation of *Hipk2* induces Wnt signaling activation and *β*-catenin nuclear localization^2^ and that HIPK2 acts as a negative regulator of the Wnt/*β*-catenin pathway. When *Hipk2* is genetically ablated, the stability of *β*-catenin is increased. Subsequently, *β*-catenin accumulates in the nucleus, thereby giving rise to an increase in Wnt target gene expression and cell proliferation.^2^ However, the role of Wnt/*β*-catenin signaling in the cerebellum is still poorly understood.

Noticeably, in an attempt to uncover the role of the Wnt/*β*-catenin pathway in the cerebellum, we observed that *β*-catenin and ubiquitin accumulate in *Hipk2*^*−/−*^ Purkinje cells. We speculate that such overexpression is probably because of a severe proteasomal dysfunction that inhibits *β*-catenin degradation, a phenomenon typically linked to neurodegenerative disorders^10, 21^ such as Parkinson's disease^22^ and spinocerebellar ataxia.^23^ Therefore, we hypothesize that *β*-catenin is ubiquitinated but not degraded in *Hipk2*^*−/−*^ Purkinje cells. Equally important, we found that mice lacking HIPK2 displayed gait, balance, and motor coordination impairment. Indeed, *Hipk2*^*−/−*^ mice performed rather poorly on beam balance and wire hanging behavioral tests. Such motor coordination defects depend mainly on a faulty cerebellar circuitry.^24, 25^ In fact, *Hipk2*^*−/−*^ mice, evaluated at postnatal day 120, an age in which the cerebellum is completely developed and HIPK2 expression is significantly increased compared with newborns, showed from the very first training session an abnormal behavior, suggesting a profound disinhibition of deep cerebellar nuclei (DCN) co-occurring with Purkinje cell loss. This is not surprising as Purkinje cells, by providing specific timing signals for movement coordination, are essential for cerebellar motor control.^26^

Our results seem to suggest that some forms of cerebellar disorders might be partly because of the lack of Hipk2 and candidate *Hipk2* as a novel gene involved in the pathogenesis of some forms of ataxia-like cerebellar disorders. This hypothesis is supported by the fact that the human *HIPK2* gene has been included in a list of genes whose expression is modified in patients affected by Friedrich's ataxia.^27^ Therefore, we hypothesize that the absence of *Hipk2* can be somehow linked to this type of cerebellar ataxia. On the other hand, we cannot rule out the possibility that among the numerous kinds of cerebellar ataxia sharing similar molecular mechanisms, others also can be linked to *Hipk2*.

In summary, several insightful findings emerged on the possible players involved in cerebellar dysfunction. In particular, we found that *Hipk2* genetic ablation has a causative role in ataxia-like cerebellar disorders. Equally important, we shed some light on the potential role of Wnt/*β*-catenin pathway in the cerebellum and on how it cooperates with Hipk2. In this instance, we observed that in Purkinje cells the lack of Hipk2 negatively interferes with Wnt/*β*-catenin proper function, giving rise to an impaired degradation of *β*-catenin – a hallmark of Parkinson's disease, spinocerebellar ataxia, and other neurodegenerative diseases. Finally, having provided support to the hypothesis that cerebellar dysfunction is associated with Hipk2 loss in Purkinje cells, this study raises the possibility that Hipk2 may be considered a potential therapeutic target for treating some forms of cerebellar ataxia.

## Materials and Methods

### RNA extraction, RT-PCR, and quantitative real-time PCR (qRT-PCR)

Total RNA was extracted from tissues with Trizol (Life Technologies, Inc., Carlsbad, CA, USA) according to the manufacturer's instructions and as previously described.^28^ qRT-PCR analysis was performed using the Quantitect Reverse transcription kit (QIAGEN, Hilden, Germany). The quantitative real-time PCR (qRT-PCR) assay was designed with the Human Probe Library system (Exiqon, Veldben, Denmark). qRT-PCR for *Hipk2* was performed using Power SYBR Green PCR Master Mix (Applied Biosystems, Foster City, CA, USA) and the following primers: mHipk2-Fw 5′-CCACATGTCAATTGCCTCAC-3′ and mHipk2-Re 5′-AGGTCATTGACTTTGGTTCAG-3′ mG6pd-Fw 5′-CAGCGGCAACTAAACTCAGA-3′ and mG6pd-Re 5′-TTCCCTCAGGATCCCACAC-3′.

The relative expression levels were calculated by using the 2-ΔΔCT method.^29^ Primers specific for the glucose-6-phosphate dehydrogenase (*G6pd*) were used for normalization of quantitative real-time PCR data.

Total RNA extraction and RT-PCR analysis were performed as previously described;^30^ PCR amplification was performed with the following primers: Hipk2-Fw 5′-GAGACACAGGCTCAAGATGG-3′ and Hipk2-Re 5′-TCTGCTCGTAAGGTAGGCTT-3′ Actin-Fw 5′-CTAAGGCCAACCGTGAAAAG-3′ and Actin-Re 5′-ACCAGAGGCATACAGGGACA-3′.

### Western blotting and immunoprecipitation assay

Different brain sections were homogenized and total protein extracts were prepared with lysis buffer RIPA as previously described.^31^ Total protein extracts were separated by SDS-PAGE and transferred onto nitrocellulose transfer membranes (Perkin Elmer, Waltham, MA, USA). Membranes were blocked with 5% BSA (bovine serum albumin protein in TBS 1% buffer, 0.02% sodium azide) and incubated with antibodies at the appropriate dilutions. The filters were incubated with horseradish peroxidase-conjugated secondary Antibodies, and the signals were detected with ECL (Thermoscientific, Waltham, MA, USA). The antibodies used for western blotting were as follows: anti-HIPK2 (Novus Biologicals, Littleton, CO, USA), anti-*β*-catenin (BD Transduction Laboratories, San Josè, CA, USA), anti-COX 2 (BD Biosciences, San Josè, CA, USA), anti-AKT (Santa Cruz, Dallas, TX, USA), anti-*β*-Tubulin (Sigma, Milan, Italy), anti-Vinculin (Santa Cruz), anti-Ubiquitin (Cell Signaling, Denvers, MA, USA), anti-Calbindin (Millipore, Darmstadt, Germany), anti-Parvalbumin (Millipore), anti-iba1 (Millipore), anti-NeuN (Millipore), and anti-GFAP (Novus Biologicals).

Immunoprecipitation experiments were performed as previously described.^28^ Briefly, cells were lysed in lysis buffer and 0.5 mg of extract was incubated with *β*-catenin antibody (BD Transduction Laboratories). Immunocomplexes were collected by centrifugation at 10 000 r.p.m. for 1 min at 4 °C and then separated by SDS-PAGE.

### Animals

*Hipk2*-null mice were generated through homologous recombination by eliminating the exon 3 coding for the entire catalytic domain at the N-terminus of the molecule, as previously reported.^13^ The correct orientation of the targeted allele was assessed by PCR analysis using a forward primer upstream of the 5′ arm and a reverse primer selected in the selection cassette.

Mice and MEFs were genotyped using the following primers to amplify either wild-type or targeted allele: Hipk2-Fw 5′-TAGTACCCAGGTGAACCTTGGAGT-3′ Hipk2wt-Re 5′-GCTTCTCTCAAACTAAAGACCACGC-3′ Hipk2KO-Re 5′-CAAAGGGTCTTTGAGCACCAGA-3′.

MEFs were isolated from 12.5 dpc embryos. After head removal, embryos were washed with PBS, incubated in trypsin 1% (Sigma) for 10 min at RT, pelleted, and then resuspended in DMEM.

### Experimental groups

Sv/129 wild-type or *Hipk2*^*−/−*^ mice were housed under diurnal lighting conditions (12 h darkness/light). Experiments were performed according to the international guidelines for animal research. The experimental protocol was approved by the Animal Care Committee of the ‘Federico II' University of Naples. As for all behavioral tests, the apparatus was wiped with 70% ethanol before and after each experiment.

### Tissue processing, immunostaining, and confocal immunofluorescence

For the histological examination, animals were placed under deep anesthesia with chloral hydrate (400 mg/kg, i.p.) and transcardially perfused with saline solution containing 0.01 ml heparin, followed by 60 ml of 4% paraformaldehyde in saline solution. The brains were then removed and postfixed overnight at +4 °C and cryoprotected in 30% sucrose in 0.1 M phosphate buffer (PB) with sodium azide 0.02% for 48 h at 4 °C.^32^ Next, they were sectioned frozen on a sliding cryostat at 30 *μ*m thickness. The sections underwent heat-induced epitope retrieval at 96 °C for 45 min in 0.01 M citrate buffer (pH 6.0). Afterward, free floating sections were incubated with PB Triton × 0.3% and blocking solution (0.5% milk, 10% FBS, 1% BSA) for 2 h. The primary antibodies were the following: mouse monoclonal anti-NeuN (1 : 1000, Millipore), mouse monoclonal anti-HIPK2 (1 : 200, kindly provided from S Soddu, Rome, Italy),^6^ rabbit polyclonal anti-parvalbumin (1 : 500, Calbiochem, Darmstadt, Germany), rabbit polyclonal anti-GFAP (1 : 500, Novus Biologicals), rabbit polyclonal anti-Calbindin D28K (1 : 300, Millipore), mouse monoclonal anti-Calbindin D28K (1 : 1000, Swant, Marly, Switzerland), rabbit polyclonal anti-*β*-catenin (1 : 2000, Abcam, Cambridge, UK), mouse monoclonal anti-Ubiquitin (P4D1) (1 : 700, Cell Signaling), rabbit polyclonal anti-BCL-2 N19 (1 : 1000 Santa Cruz), rabbit polyclonal anti-BAX (1 : 1000 Cell Signaling), rabbit polyclonal anti-BCL-XL (1 : 1000 Cell Signaling). The sections were incubated with the corresponding florescent-labeled secondary antibodies (Alexa 488/Alexa 594-conjugated antimouse/antirabbit IgGs). Nuclei were counterstained with Hoechst. Images were observed using a Zeiss LSM510 META/laser scanning confocal microscope (Zeiss, Oberkochen, Germany). Single images were taken with an optical thickness of 0.7 m and a resolution of 1024 × 1024. In double-labeled sections, the pattern of immunoreactivity for both antigens was identical to that seen in single-stained material. Control double-immunofluorescence staining entailed the replacement of the primary antisera with normal serum (data not shown). To minimize a possible crossreactivity between IgGs in double immunolabeling experiments, the full complement of secondary antibodies was maintained but the primary antisera were replaced with normal serum or only one primary antibody was applied (data not shown). In addition, the secondary antibodies were highly pre-adsorbed to the IgGs of numerous species. Tissue labeling without primary antibodies was also tested to exclude autofluorescence. No specific staining was observed under these control conditions, thus confirming the specificity of the immunosignals. Standard 3,3′-diaminobenzidine (DAB) staining was employed on sagittal step serial sections using antibody directed against calbindin protein.

### Cell-counting analysis

The number of calbindin cells was determined in the cortex of the cerebellum of wild-type and *Hipk2*^*−/−*^ mice at post natal days 21 and 120, by manual counting at × 10 magnification. The consecutive and identical sections were selected starting from bregma −5.80 mm, interaural –2.00, from both wild-type and *Hipk2*^*−/−*^ mice.

Only calbindin cells with clearly visible cell bodies and profiles were counted. Three mice per group were included in the studies, and six slices from every mouse were analyzed.^33^

### Beam walking test

Sensory-motor coordination was tested using balance beams (45 cm length; 30% incline). Each mouse was given three trials per beam for three consecutive days. Latency to traverse the beam was scored and averaged. Failure to traverse the beam during the allotted time terminated the trial and the maximum time (180 s) was measured.^34^

### Wire hanging test

The mice were held so that only their forelimbs were able to reach an elevated bar (50 cm in length, 2.5 mm diameter, 40 cm above the floor). The mice were scored as follows: 0: fell off; 1: hung onto the wire by both forepaws; 2: hung onto the wire by both forepaws while also attempting to climb onto the wire; 3: hung onto the wire by both forepaws plus one or both hindpaws around the wire; 4: hung onto the wire by all four paws while having its tail wrapped around the wire; and 5: managed to escape.^35^ Each mouse received three opportunities for three consecutive days, and latency to fall off the wire was also measured up to a maximum of 60 s. Intertrial intervals for all animals were 10 min long.

### Statistical analyses

Data were first analyzed by ANOVA followed by Bonferroni's test as the *post hoc* test using GraphPad Prism 5.0 (La Jolla, CA, USA) (Figures 1A–C and Figure 2A). Student's *t-*test was used for cell counting and for densitometric analysis (Figures 2B, 3A, 3C and 3D, 5A, 6A and 6B and Supplementary Figures 2a–d). One-way or two-way ANOVA, followed by the Tukey's HSD test, was used for behavioral analysis (Figure 7), and the nonparametric Kruskal–Wallis test was used for the wire hanging tests (Figure 7a). Details of the statistical analyses are described in the figure legends.

## Abbreviations

CA: cornu ammonis
CNS: central nervous system
COX-2: cyclooxygenase-2
DCN: deep cerebellar nuclei
GFAP: glial fibrillary acid protein
HIPK: homeodomain-interacting protein kinase
TGF: transforming growth factor
