# Supplementary material for: Genetic ablation of homeodomain-interacting protein kinase 2 selectively induces apoptosis of cerebellar Purkinje cells during adulthood and generates an ataxic-like phenotype
Source: Cell Death Dis. 2015 Dec 3;6(12):e2004–. doi: 10.1038/cddis.2015.298 (PMC4720876; doi:10.1038/cddis.2015.298)
Supplement: Supplementary Figure Legends [file cddis2015298x1.doc]

**Supplementary Figure S1** Lack of HIPK2 expression in Hipk2-KO mice. (A) PCR analysis of Hipk2 gene on DNA extracted from wild-type, heterozygous Hipk2^+/-^, and homozygous Hipk2^-/-^ mice. (B) Left panel, RT-PCR expression analysis of Hipk2 gene in wild-type and Hipk2-/- MEFs at passage 3. Actin gene expression was used as control. Right panel, Western blot analysis of HIPK2 protein in wild-type and Hipk2-/- proteins extracted from MEFs at passage 3. Vinculin was used as loading control. (C) RT-PCR expression analysis of Hipk2 gene in wild-type and Hipk2-/- liver tissue. Actin was used as loading control

**Supplementary Figure S2** *Hipk2^-/-^* mice showed no alteration in microglia and granular cells of cerebellum. Total cellular extracts from cerebellum of wild-type and *Hipk2^-/-^* mice were analyzed by ~~W~~Western ~~B~~blot using the indicated antibodies. (**A**) The graph shows densitometric analysis from three independent experiments performed with anti-Iba1 antibodies, and one indicative experiment is shown. β-tubulin was used for normalization. (**B**) Densitometric analysis from three independent experiments performed with anti-NeuN antibodies; one indicative experiment is shown**.** AKT was used for normalization. Data represent the mean ± SD
