# Supplementary figures and images for: Genetic ablation of homeodomain-interacting protein kinase 2 selectively induces apoptosis of cerebellar Purkinje cells during adulthood and generates an ataxic-like phenotype
Source: Cell Death Dis. 2015 Dec 3;6(12):e2004–. doi: 10.1038/cddis.2015.298 (PMC4720876; doi:10.1038/cddis.2015.298)

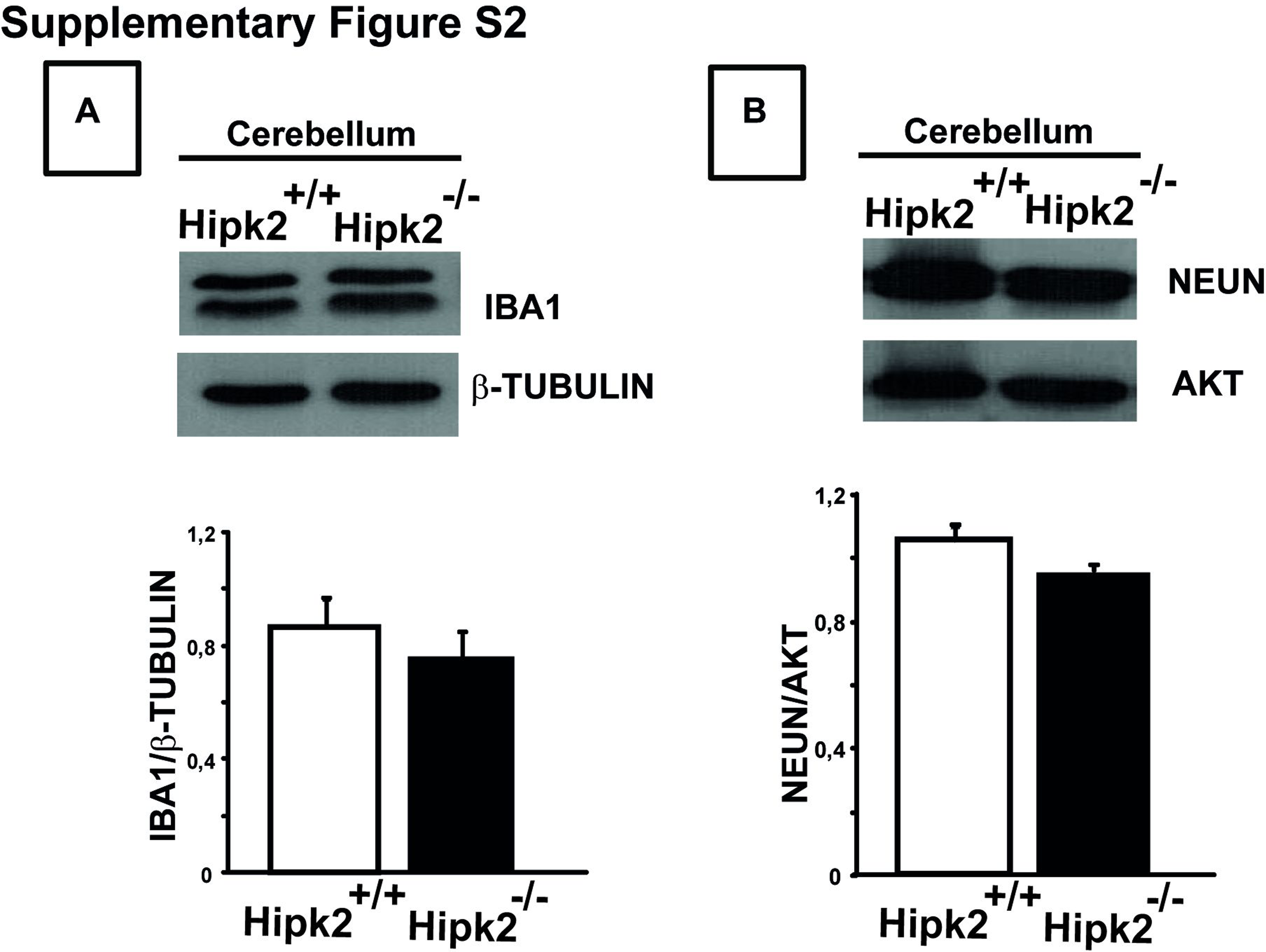

Supplement: Supplementary Figure S2 [file cddis2015298x3.tif]
